# A novel mutation in calcium-sensing receptor gene associated to hypercalcemia and hypercalciuria

**DOI:** 10.1186/1472-6823-14-81

**Published:** 2014-10-07

**Authors:** Eugenio Mastromatteo, Olga Lamacchia, Michela Rosaria Campo, Antonella Conserva, Filomena Baorda, Luigia Cinque, Vito Guarnieri, Alfredo Scillitani, Mauro Cignarelli

**Affiliations:** Unit of Endocrinology and Metabolic Diseases, Department of Medical and Surgical Sciences, University of Foggia, Italy, Viale Pinto, 1, 71122 Foggia, Italy; Medical Genetics Service IRCCS “Casa Sollievo della Sofferenza” Hospital viale Padre Pio, 71013 San Giovanni Rotondo, (FG) Italy; Unit of Endocrinology and Metabolic Diseases IRCCS “Casa Sollievo della Sofferenza” Hospital viale Padre Pio, 71013 San Giovanni Rotondo, (FG) Italy

**Keywords:** CaSR gene, Hyperparathyroidism, FHH, Hypercalcemia, Hypercalciuria, Hypocalciuria

## Abstract

**Background:**

Familial Hyperparathyroidism (HPT) and Familial benign Hypocalciuric Hypercalcemia (FHH) are the most common causes of hereditary hypercalcemia. FHH has been demonstrated to be caused by inactivating mutations of calcium-sensing receptor (CaSR) gene, involved in PTH regulation as well as in renal calcium excretion.

**Case presentation:**

In two individuals, father and son, we found a novel heterozygous mutation in CaSR gene. The hypercalcemia was present only in father, which, by contrast to the classic form of FHH showed hypercalciuria (from 300 to 600 mg/24 h in different evaluations) and a Calcium/Creatinine ratio of 0.031, instead of low or normal calciuria (<0.01 typical finding in FHH). His son showed the same mutation in CaSR gene, but no clinical signs or hypercalcemia although serum ionized calcium levels were close to the upper limit of normal values (1.30 mmol/L: normal range: 1.12-1.31 mmol/L). Sequence analysis revealed a point mutation at codon 972 of CaSR gene (chromosome 3q), located within cytoplasmic domain of the CaSR, that changes Threonine with Methionine. The father was treated with Cinacalcet 90 mg/day, with a decrease of total serum calcemia from an average value of 12.2 mg/dl to 10.9 mg/dl.

**Conclusion:**

This is a case of a novel inactivating point mutation of CaSR gene that determines an atypical clinical presentation of FHH, characterized by hypercalcemia, hypercalciuria and inadequate normal PTH levels. Functional assay demonstrated that the 972 M variant influenced the maturation of the protein, in terms of the post-translational glycosylation. The impairment of the receptor activity is in keeping with the specific localization of the 972 residue in the C-terminal tail, assigned to the intracellular signalling, that on the basis of the our findings appears to be differently modulated in parathyroid gland and in kidney.

## Background

CaSR gene (chr. 3q13.3-21) encodes for a protein of 1078 aminoacids present in the plasma membrane as a dimer. CaSR is a member of the G-protein coupled receptors and its structure has 3 different domains [1, 2]. The extracellular domain (612 aminoacids) binds extracellular calcium through its multiple negative charges allowing the CaSR to function as a sensitive detector of extracellular calcium; the transmembrane part (250 aminoacids) has 7 membrane-spanning domains; the intracellular tail (216 aminoacids) interacts with the G-proteins and filamin A to translate within the cells the signal produced by the extracellular calcium binding [3, 4]. Through these and other pathways, CaSR may influence cell function, especially PTH secretion from parathyroid cells, but also cell proliferation and gene expression [5, 6]. This process takes place mainly in parathyroid and kidney tubular cells, regulating calcium concentrations in extracellular fluid. In the kidney, the CaSR performs different tasks depending on the various tubular segments in which it is located [7]. It is expressed on the luminal membrane of the proximal tubular cells where it senses the increase in calcium luminal concentrations and inhibits cAMP production induced by PTH [8, 9]. CaSR is expressed on the basolateral membrane of the thick ascending limb of Henle loop [9], where modulates the electric gradient generated by sodium-potassium reabsorption and potassium recycling, inhibiting the sodium-potassium-chloride carrier activity. Therefore, after an increase in serum calcium, CaSR decreases the potential, eventually supporting calcium reabsorption and promoting calcium excretion [5]. In the distal convoluted tubule, CaSR is located on the basolateral membrane of tubular cells where reduces the active calcium reabsorption by interfering with the calcium pump function through a phospholipase C dependent mechanism [10]. In adults, inactivating mutations of CaSR gene are found in FHH, an autosomal dominant disease characterized by moderate but significant hypercalcemia, accompanied by few symptoms [11, 12], with inappropriately normal serum PTH levels, and by a low or normal urinary calcium levels [12–14] with histologically normal parathyroid glands. The condition does not require treatment, and responds poorly to parathyroidectomy. FHH should be distinguished from primary HPT, in which the elevated serum and urinary calcium levels are normalized by successful parathyroid surgery. Familial primary HPT is inherited as autosomal dominant mutation either as the single lesion (isolated HPT form) [15] or in the context of multiple endocrine neoplasia (MEN) type 1 or 2A [16] and the HPT-jaw tumor syndrome [17].

## Case presentation

A 68-year-old male patient presented with slight fatigue and intermittent polyuria. His family history was positive for osteoporosis, negative for fractures and nefrolitiasis. In childhood he was subjected to a jaw cementoma asportation. He underwent several surgical procedures because of recurrent nefrolitiasis since 25 years. He has also been on hypertension treatment since 30 years. At presentation we observed arterial pressure of 120/70 mmHg and pulse rate of 70 beats/min with his habitual antihypertensive therapy (Losartan 50 mg/day). Physical examination was unremarkable: weight 75 Kg, height 173 cm, body mass index 25.1 Kg/m^2^_._ Total serum calcium level at first evaluation was 11.6 mg/dl, with a PTH improperly normal (25.7 pg/ml). Low values of serum 25-hydroxy-Vitamin D_3_ was also observed (16 ng/ml). During the following visits we found hypercalciuria (from 300 to 600 mg/24 h in different evaluations) and a Ca/Cr ratio of 0.031. Glycemia, TSH, prolactin, IGF-1, urine metanephrines levels were in normal range. We found higher values of serum Anti-Parietal-Cells-Antibodies (APCA), suggestive for chronic autoimmune gastritis. Laboratory results are shown in Table 1.

**Table 1:** **Laboratory results (follow up period)**

	Patient I:1			
	First values	1 year follow-up (after cinacalcet)	2 years follow-up (no specific therapy)	4 years follow-up (no specific therapy)
s-Ca total (correct) *(n.v. 8.8-10.6)*	11.6 mg/dl	10.9 mg/dl	12.96 mg/dl	11.8 mg/dl
s-PO4 *(n.v. 2.7-4.5)*	3 mg/dl	2.5 mg/dl	2.2 mg/dl	2.4 mg/dl
s-Creatinine *(n.v. 0.2-1.2)*	1.08 mg/dl	1.12 mg/dl	1.31 mg/dl	1.19 mg/dl
s-PTH *(n.v. 5–40)*	25.7 pg/ml	33 mg/dl	19.4 mg/dl	25.4 mg/dl
25(OH)vitamin D *(n.v. 30–100)*	16 ng/ml	16 ng/ml	40.7 ng/ml	-
U Ca *(n.v. < 4 mg/Kg)*	-	658 mg/24 h	315 mg/24 h	325 mg/24 h
Cr Clearance (*n.v.* 70*–*120 ml/min)	-	67 ml/min	55 ml/min	61 ml/min
Ca/Cr ratio	-	0.041	0.031	0.030

Neck echography, NMR and 99mTc-sesta MIBI tomoscintigraphy were negative for parathyroids glands images. Abdomen echography and CT showed bilateral polycystic kidney (maximum diameter 8.5 cm). Bone density scan (DXA) showed normal bone mineral density (Femur T-score 1.07, Lumbar T score 1.3). Recent orthopantomography was negative for jaw lesions.

**Figure 1:**
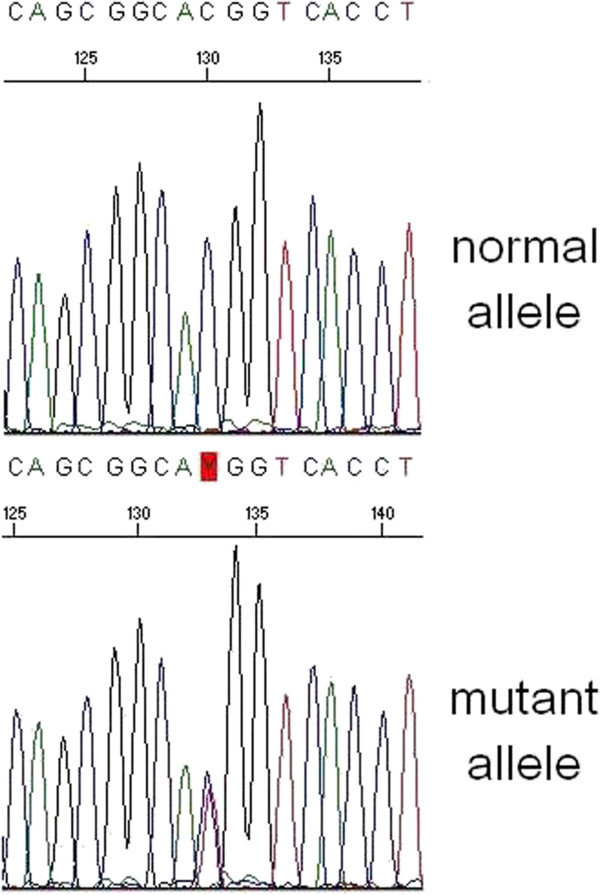
**Elechtropherogram showing the presence of the c.2915C > T variant in heterozygosity.** On the top the control, on the bottom the mutated sequence.

**Figure 2:**
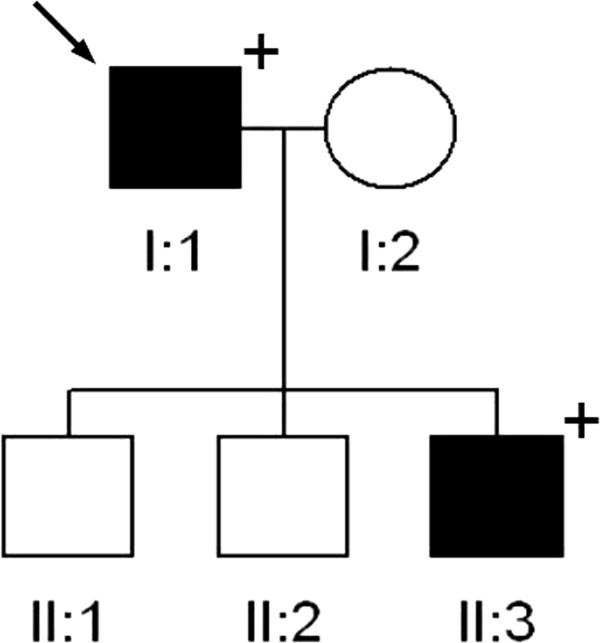
**Family tree of family under study.** Black box indicates the presence of PHPT, the arrow the presence of the mutation (see text for clinical details).

The patient was treated for 7 months with Cinacalcet 90 mg/day, since at that time we ignored the existence of the CaSR mutation. However, although Cinacalcet is not indicated for FHH treatment, we obtained a slight decrease of calcemia, but the therapy was stopped because of side effects appearance (hypotension, nausea). Subsequently, he refused other specific therapies. During a 4 years follow-up period hypercalcemia, hypercalciuria and normal levels of PTH persisted. A heterozygous mutation in exon 7 of the CaSR gene was found, predicting a p.T972M amino acid substitution in cytoplasmic tail of CaSR (Figure 1). The same mutation we found in one of his three screened sons, a 41-year-old men (Figure 2), who is currently asymptomatic and shows serum ionized calcium level of 1.30 mmol/L, close to the upper normal limit (n.v. 1.12-1.31), normal calciuria (195 mg/24 h) and PTH, but also a 25 hydroxy-Vitamin D_3_ deficiency (12.3 ng/ml) (Table 2).

**Table 2:** **Laboratory results (comparison among father and his affected son at genetic evaluation time)**

	Patient I:1	Patient’s affected son II:3
Age at diagnosis	70 years old	41 years old
s-Ca total *(n.v. 8.8-10.6)*	12.96 mg/dl	10.3 mg/dl
S-Ca^++^ (*n.v. 1.12-1.31*)	1.62 mmol/l	1.30 mmol/l
s-PO4 *(n.v. 2.7-4.5)*	2.2 mg/dl	3.3 mg/dl
s-PTH *(n.v. 5–40)*	19.4 pg/ml	23.5 pg/ml
25(OH)vitamin D *(n.v. 30–100)*	40.7 ng/ml	12.3 ng/ml
U Ca *(v.n. <4 mg/kg)*	315 mg/24 h	195 mg/24 h
Cr Clearance (*n.v.* 70*–*120 ml/min)	55 ml/min	107 ml/min
Ca/Cr ratio	0.031	0.012

**Figure 3:**
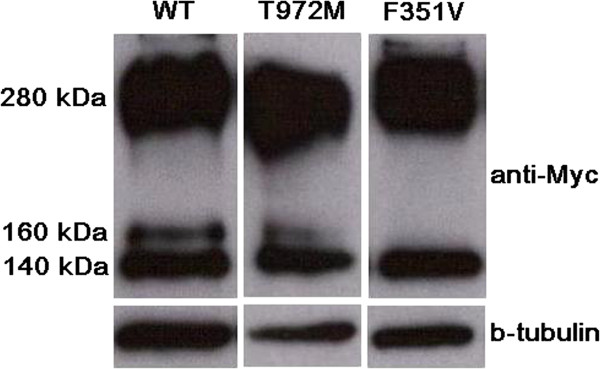
**Western blot on protein extract.** CaSR protein possesses different levels of maturation in terms of post-translational modifications: with respect to the WT, the 972 M mutant showed underexpressed the fully mature glycosylated form of 160 kDa, while the other two bands (the immature not-glycosylated 140 kDa form and the dimers band of 280 kDa) showed a normal pattern.

**Figure 4:**
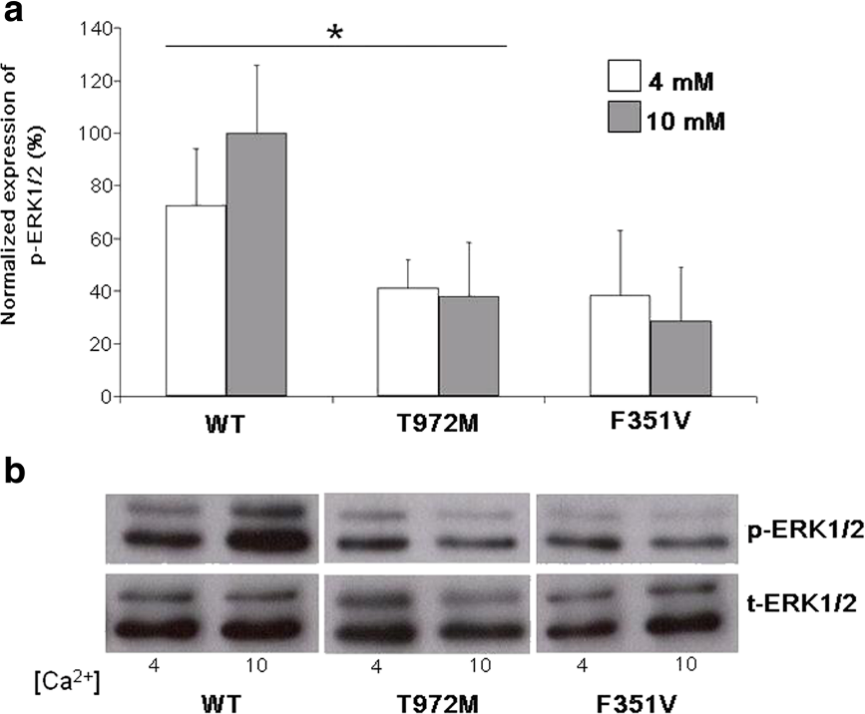
**CaSR signaling activity measured as the ratio between the level of phosphorylated and total p42/44 proteins. a**: compared with the WT for both the Ca^2+^ concentrations, the 972 M variant induced a lower level of phosphorylation, similarly to the activity mediated by the inactivating control (F351V). Values were normalized with respect to the WT at 10 mM. Data are reported as mean ± SEM of three replicate experiments. * = p <0.05 compared with the WT; **b**: a representative western blot was shown.

We also tested the mutation by either qualitative assay in order to investigate the protein conformation effect or by quantitative assay to verify in addition the alteration of protein function. In our functional assays on WT and mutant CaSR proteins, we showed that, with respect the WT, the 972 M variant is slightly underexpressed as far as the 160 kDa glycosylated form (Figure 3). The signaling activity of the 972 M mutant receptor, compared to the WT, resulted strongly impaired even at higher Ca^++^ concentration (10 mM), showing a pattern similar to the inactivating mutant control (Figure 4).

## Methods

Calcium, phosphorous and creatinine were measured by automated laboratory methods. The urinary calcium and creatinine measurements were carried out on spot and on 24 h collection. PTH (Liaison 1–84 PTH, reference values 5–40 pg/ml) and serum 25-OH-cholecalciferol were determined by chemiluminescence immunoassay (Diasorin-Liaison XL). Neck tomoscintigraphy was performed by injection of 99Tc-sesta MIBI.

### Molecular analysis of CaSR gene

DNA was extracted from peripheral blood leukocytes using standard protocol. Signed informed consent was obtained from all the subjects and the protocol was approved by local ethical committee of IRCCS “Casa Sollievo della Sofferenza” Hospital (San Giovanni Rotondo). Genetic screening of CaSR gene was performed by PCR amplification and direct sequencing of all the 7 exons (12 amplicons) including exon-intron boundaries as previously reported [18].

### cDNA expression vectors and mutagenesis

The 972 M variant was introduced in a Myc tagged human Wild Type (WT) CaSR cDNA expressing pCDNA3.1 vector: briefly, mutagenesis reaction was carried out with the following primers (p.T972M, For: 5’ -catctttggcagcggcaTggtcaccttctcactga- 3’ and Rev: 5’ -tcagtgagaaggtgaccAtgccgctgccaaagatg- 3’, mutated base is in capital). One microlitre of Dpn1 (New England Biolabs) digested the parental DNA while 3 ul of reaction was used to transform E.coli 5-alpha chemically competent cells (Lucigen). The mutated clone was identified by colony PCR and sequencing while midiprep performed with Plasmid Midi Kit (QIAGEN).

### Cell culture and transfection

Human Embrionic Kidney (HEK293, ECACC) cells were cultured in DMEM/F12 (Sigma) supplemented with FBS 10% (Sigma) and penicillin/streptomycin 5% (SIGMA) at 37°C with CO_2_ 5%.

### Western blot on crude lysate protein extracts

200 thousands HEK293 cells were seeded in 12 wells plates and transfected with Myc-tagged CaSR WT and mutant vectors (T972M and F351V, this latter as inactivating control) in triplicate. 48 hours after transfection, total cell proteins were extracted in RIPA buffer [150 mMNaCl, 50 mM Tris–HCl, 1%, Nonidet P-40, 0.1% sodium dodecyl sulfate (SDS), 0.5% sodium deoxycholate, pH 8.0] supplemented with one tablet/10 mL of PhosStop and Complete EDTA, phosphatase and protease inhibitors (both from Roche). 50 ug of proteins were loaded onto a 8% SDS polyacrylamide gel, electrotransferred to PVDF membrane (Millipore, Billerica, MA), blotted overnight at 4°C with rabbit anti Myc monoclonal antibody (Cell Signaling Technology, 1:800 in blocking solution) and for 1 h at room temperature with the horseradish peroxidase-conjugated goat anti-rabbit IgG antibody (Santa Cruz Biotechnology) as secondary antibody. Membrane was stripped with Re-Blot Solution (Millipore) and re-blotted with b-tubulin rabbit monoclonal antibody (Cell Signaling Technology, 1:1000 in blocking solution) as loading control.

### MAP Kinase assay

750 thousand HEK293 cells were seeded in 6 wells plates and transfected in 6 replicates with WT or mutant vectors using Lipofectamine 2000 (Invitrogen). After 24 h cells were starved with DMEM/F12 without Mg^++^ and with minimal Ca^++^ concentration (0,5 mM final). 48 h after the transfection, cells were stimulated in triplicates for each vector with 4 or 10 mM CaCl_2_ for 5 min. Whole-cell extracts were made with RIPA buffer supplemented with cocktail inhibitors as above described. 80 ug of proteins were loaded onto a 12% SDS-PAGE and analyzed for expression of phosphorylated and total ERK1/2 proteins by immunoblotting with rabbit Phospho p42/44 and rabbit Total p42/44 antibodies, respectively (Cell Signaling Technology) according to the manufacturer’s protocol. Secondary antibody was as above described. The Image J-National Institutes of Health image processing program (http://rsb.info.nih.gov/ij/) was used for signal densitometry by determining the ratios of the phosphorylated to nonphosphorylated ERK1/2 signals at various extracellular calcium concentrations and then normalized to the ratio of phosphorylated to unphosphorylated ERK1/2 at 10 mM [Ca^++^].

### Statistical analysis

Results are expressed as mean and standard deviation derived from triplicates experiments. A p-value < 0.05 was considered for statistical significance.

## Discussion

Familial hyperparathyroidism is a wide spectrum of disorders including multiple endocrine neoplasia types 1 (MEN 1) and 2A, hyperparathyroidism-jaw-tumor syndrome (HPT-JT), familial hypocalciuric-hypercalcemia (FHH) and familial isolated hyperparathyroidism (FIHP) [16, 17]. We report the case of FHH (an adult men and his son) affected by a novel mutation in CaSR gene. The screening of the whole coding sequence of the CaSR gene led to the identification of a novel variant of the exon 7, namely c.2915C > T, p.T972M (Figure 1) in heterozygosity. This variant was absent on the public CaSR database (http://www.casrdb.mcgill.ca/; last access: may 2014) nor on either the Mutation Discovery and 1000 Genomes databases (http://www.mutationdiscovery.com/md/MD.com/home_page.jsp; http://www.1000genomes.org/; last access: may 2014), thus ruling out the possibility that it was a rare polymorphism. The segregation analysis enlarged to the available first degree relatives (Figure 2) allowed the identification of one carrier (II:3), the affected patient’s son, a 41-year-old men, who is currently asymptomatic and shows serum ionized calcium reaching upper value (1.30 mmol/L) of normal range (1.12-1.31 mmol/L), normal calciuria and PTH. This point mutation, inherited in heterozygosity and implicated in inactivating CaSR, leads to a loss of function of the receptor, with evidence, in the father, of hypercalcemia and unsuppressed PTH levels. In classic form of this disorder, there is normal or hypocalciuria (Ca/Cr ratio <0.01), as expected when there is a point mutation confined in extracellular and transmembrane domains of CaSR.

In our subject, instead, there is evidence of hypercalciuria, in line with Carling *et al*. study [19], that identified a family with a heterozygous inactivating mutation of the cytoplasmic domain of the CaSR, not far from that of the patients in our study, associated with hypercalcemia, hypercalciuria and unsuppressed PTH. In that family, parathyroid surgery was performed in seventeen kindreds and in some cases, hyperplasia and nodule formations interpreted as single adenoma were found. By contrast to these previous reports, no image related to parathyroids hyperplasia or adenoma was detected in both subjects. The presence of polycystic kidney and jaw-cementoma in childhood can contribute to ambiguity in distinguishing an atypical form of FHH from HPT-JT. The absence of parathyroid adenoma, no visible jaw-cementoma in the recent orthopantomography and CaSR gene mutation, not associated to HPT, likely exclude HPT-Jaw tumor syndrome. In our functional assays on WT and mutant CaSR proteins, the western blot, performed on protein crude lysates extracted from cells transfected with WT and mutants CaSR vectors, showed that, with respect to the WT, the 972 M variant is slightly underexpressed as far as the 160 kDa glycosylated form. Instead either the immature not-glycosilated 140 kDa and the higher 280 kDa band suggestive of the CaSR dimers, appeared similar to the WT (Figure 3). The N-glycosylation at eight sites (N-90, N-130, N-261, N-287, N-446, N-468, N-488, and N-541) of the human CaSR have been proved to be important for a proper cell surface expression [20, 21]. The process takes place in the ER and the fully glycosylation ends in the Golgi. So far several CaSR mutations not specifically affecting the 8-N have been functional tested and in many cases they showed a decrease of the expression of the 160 kDA band [22, 23]. In these cases an impairment of the trafficking from the ER towards the Golgi is hypothesized, rather than a defect in the N-glycosylation process *per se*, even if in both the cases the final effect is a decrease of the cell surface expression [24]. With regard the signaling activity of the mutant receptor, compared to the WT, the 972 M mutant resulted strongly impaired even at higher Ca^++^ concentration (10 mM), with a lower phosphorilation rate of ERK 1/2, showing a pattern similar to the inactivating mutant control (Figure 4).

## Conclusions

This is a case of a novel inactivating point mutation of CaSR gene that determines an atypical clinical presentation of FHH with hypercalciuria. In the functional assays we showed that the 972 M variant influenced the maturation of the protein, in terms of the post-translational glycosylation. The impairment of the receptor activity is in keeping with the specific localization of the 972 residue in the C-terminal tail, which is assigned to the intracellular signaling. It seems that this CaSR mutation influences the intracellular signaling in parathyroid cells but not in kidney, suggesting the existence in these two sites of distinct intracellular pathways. The reported case may likely contribute to enlarge the expanding clinical spectrum of CaSR inactivating mutations.

## Consent

Written informed consent was obtained from the patient for publication of this Case Report and any accompanying images. A copy of the written consent is available for review by the Editor of this journal.

## Abbreviations

HPT: Familial hyperparathyroidism
FHH: Familial benign hypocalciuric hypercalcemia
CaSR: Calcium-sensing receptor
MEN: Multiple endocrine neoplasia
APCA: Anti-parietal-cells-antibody
DXA: Bone density scan
HPT-JT: Hyperparathyroidism-jaw-tumor syndrome
FIHP: Familial isolated hyperparathyroidism
WT: Wild type.
